# Implementation Science in Pediatric Critical Care – Sedation and Analgesia Practices as a Case Study

**DOI:** 10.3389/fped.2022.864029

**Published:** 2022-07-04

**Authors:** Youyang Yang, Alon Geva, Kate Madden, Nilesh M. Mehta

**Affiliations:** Department of Anesthesiology, Critical Care and Pain Medicine, Boston Children’s Hospital and Harvard Medical School, Boston, MA, United States

**Keywords:** sedation, analgesia, pediatric critical care, implementation science, barriers

## Abstract

Sedation and analgesia (SA) management is essential practice in the pediatric intensive care unit (PICU). Over the past decade, there has been significant interest in optimal SA management strategy, due to reports of the adverse effects of SA medications and their relationship to ICU delirium. We reviewed 13 studies examining SA practices in the PICU over the past decade for the purposes of reporting the study design, outcomes of interest, SA protocols used, strategies for implementation, and the patient-centered outcomes. We highlighted the paucity of evidence-base for these practices and also described the existing gaps in the intersection of implementation science (IS) and SA protocols in the PICU. Future studies would benefit from a focus on effective implementation strategies to introduce and sustain evidence-based SA protocols, as well as novel quasi-experimental study designs that will help determine their impact on relevant clinical outcomes, such as the occurrence of ICU delirium. Adoption of the available evidence-based practices into routine care in the PICU remains challenging. Using SA practice as an example, we illustrated the need for a structured approach to the implementation science in pediatric critical care. Key components of the successful adoption of evidence-based best practice include the assessment of the local context, both resources and barriers, followed by a context-specific strategy for implementation and a focus on sustainability and integration of the practice into the permanent workflow.

## Introduction

Optimal sedation and analgesia (SA) management are critical components of care in the pediatric intensive care unit (PICU) and an essential piece of the ICU Liberation ABCDEF Bundle (1, 2). Sedative and analgesic medications are utilized in an effort to ensure safety and tolerance of the variety of invasive therapies necessary during care of the critically ill patient. Recent studies demonstrated that the long-term harm from some of these SA medications has highlighted the importance of generating high-quality evidence to guide best practices. The use of benzodiazepines, one of the mainstays of pediatric sedation management, is associated with ICU delirium and worse patient outcomes (3, 4). Overall, safe alternatives to benzodiazepines are limited, and there is a paucity of studies that could guide evidence-based recommendations for SA practices in the PICU. Furthermore, the implementation of evidence-based or consensus-driven SA practices may be impeded due to challenges related to patient heterogeneity, barriers in local culture, weak evidence of improved patient-related outcomes, and a lack of clear implementation strategies. In this narrative mini-review, we examined 13 studies related to the implementation of SA protocols in PICUs, their study design, implementation strategies, and study outcomes. We then discussed barriers to successful implementation, a few select implementation tools, and proposed future directions for the role of implementation science (IS) in successful adoption of evidence-based SA protocols.

## Sedation and Analgesia Regimens in the Pediatric Intensive Care Unit – A Summary of Recent Evidence

Several studies have examined the impact of SA regimens on clinical outcomes in critically ill infants and children over the past decade (Table 1) (5–16). Most of these studies were single-center studies conducted in tertiary or quaternary PICUs, i.e., a mix of cardiac, medical, and surgical patients.

**TABLE 1:** Summary of recent articles examining SA interventions in pediatric ICUs.

	Authors (year), Setting	Design	Intervention (target phase)	SA regimen (medications)	Implementation	Outcome
1	Deeter et al. (7) Tertiary medical-surgical-cardiac PICU	Retrospective cohort study	Nurse-driven SA protocol (initiation, titration, wean)	1st: morphine, lorazepam 2nd: fentanyl, dilaudid, dexmedetomidine	- 1 h small group training - Daily auditing of compliance - Bedside support for first week	- Reduced duration of midazolam, morphine and lorazepam infusions - Fewer days of MV (not statistically significant)
2	Curley et al. (18) 31 United States PICUs	Unblinded multicenter cluster-randomized clinical trial	SA protocol, ERT, weaning protocol (initiation, titration, wean)	1st: morphine, midazolam 2nd: fentanyl, dexmedetomidine, propofol, clonidine, pentobarbital, ketamine	- Discipline-specific education (slide packages, pocket reminder cards, bedside booklets) - Completion of discipline-specific, scenario-based post-test	- Fewer pressure ulcers - Fewer days of opioid - Exposure to less sedative classes - Greater percentage of days with pain and agitation - No change in MV duration
3	Neunhoeffer et al. (11) Medical-surgical-cardiac PICU	Pre-post implementation study	Nurse-driven SA protocol (initiation, titration, wean)	1st: morphine or fentanyl, midazolam	- Education presentations to nursing - Bedside training with experienced study-nurse - Local nursing champions	- Reduced incidence of withdrawal - Reduced total doses of opioids and benzodiazepines
4	Neunhoeffer et al. (12) Medical-surgical-cardiac PICU	Pre-post implementation study	Nurse-driven SA and withdrawal symptoms-based protocol (initiation, titration, wean)	1st: fentanyl, midazolam 2nd: clonidine, melatonin, chloral hydrate	- Education presentations to nursing - Bedside training with experienced study-nurse - Local champions available daily	- Reduced total daily dose of benzodiazepines - Reduced rate of withdrawal symptoms - No change in PICU LOS, MV duration or total daily dose of opioids
5	Dreyfus et al. (10) Medical-surgical PICU	Pre-post implementation study	Nurse-driven SA protocol (initiation, titration, wean)	1st: sufentanil 2nd: midazolam, ketamine	- 1 h training sessions - Local champions available daily	- Reduced MV duration for surgical patients - Increased COMFORT-B scores per day
6	Gaillard-Le Roux et al. (16) Medical-surgical PICU	Pre-post implementation study	Nurse-driven SA protocol (initiation, titration)	1st: midazolam, morphine or sufentanil 2nd: ketamine, clonidine	- Visual displays of protocol - Staff trainings	- No change in MV duration overall, but appeared decreased in patients older than 12 months - No difference in daily drug dose - Increased comfort assessments
7	Larson and McKeever (15) Tertiary medical-surgical-cardiac PICU	Retrospective chart review	Nurse-driven SA protocol (initiation, titration)	1st: morphine, clonidine 2nd: midazolam Other: fentanyl, dexmedetomidine, propofol	(not described)	- Increase in pain assessments - Reduction in midazolam administration - Increased duration of MV
8	Amirnovin et al. (9) Tertiary cardiac PICU and acute ward	Pre-post implementation study	Opioid and benzodiazepine protocol (wean)	1st: methadone, hydromorphone, lorazepam	- Educational lectures - Mandatory post-education testing - “Just-in-time” education	- Shorter duration of opioids and benzodiazepines - Decrease in withdrawal occurrence - Reduction in hospital LOS
9	Donnellan et al. (8) Tertiary cardiac PICU	QI PDSA cycles with SPC charts	Comfort-guided SA protocol (initiation, titration, wean)	1st: morphine, dexmedetomidine 2nd: lorazepam	- Bedside review with nurse prior to patient admission	- Decreased opioid infusion rates - Near-eliminated benzodiazepine infusions - No change in MV duration or PICU LOS
10	Sanavia et al. (14) Tertiary PICU	Prospective observational study	SA drug rotation protocol (initiation, titration, wean)	1st: fentanyl, midazolam, clonidine rescue 2nd: ketamine, propofol, metamizole rescue 3rd: remifentanil, midazolam, clonidine rescue 4th: metamizole, dexmedetomidine, morphine rescue	- Training sessions and review sessions for all PICU staff over 15 days	- Lower incidence of withdrawal syndrome - Shorter PICU LOS - Less time of opioid, benzodiazepine and propofol infusion
11	Hanser et al. (5) Tertiary cardiac PICU	Retrospective observational study	Nurse-driven SA protocol (initiation, titration, wean)	1st: morphine, midazolam 2nd: clonidine, melatonin, chloral hydrate	(not described)	- Reduced PICU LOS - Reduced midazolam and morphine exposure - No change in MV duration
12	Yang et al. (6) Quaternary PICU	Pre-post implementation study	SA protocol (initiation, titration)	1st: morphine, dexmedetomidine 2nd: lorazepam or midazolam	- Virtual educational modules with mandatory post-education test - Visual aids, educational lectures, bedside teaching	- Reduced dose and duration of midazolam - No change in PICU LOS or MV duration
13	Shildt et al. (13) Quaternary PICU	Retrospective cohort study	SA protocol (initiation, titration)	1st: morphine or fentanyl, dexmedetomidine 2nd: hydromorphone 3rd: midazolam or lorazepam	- Multidisciplinary training sessions	- Decreased opioid withdrawal and need for methadone - Decreased MV duration - Decreased PICU and hospital LOS

*PICU, pediatric intensive care unit; SA, sedation-analgesia; LOS, length of stay; MV, mechanical ventilation; ERT, extubation readiness testing; QI, quality improvement; PDSA, plan-do-study-act; SPC, statistical process control.*

A variety of outcomes were considered in these studies. Exposure to SA medications was the most common outcome assessed after the implementation of an SA regimen. Of the 13 studies included in this review, 12 (92%) studies had interventions examining initiation and titration of SA medications and 8 (62%) included a sedation/analgesia weaning protocol. In total, 11 studies (85%) demonstrated a significant reduction in either duration or total dose of opiates, benzodiazepines, or another sedative of interest. Patient-centered benefits were explored in some of the studies with variable results (5, 7, 9–14). Mechanical ventilation duration was a study outcome in 12 (92%) of the 13 studies; it was statistically significantly decreased in 2 (17%) studies, unchanged in 9 (75%), and increased in 1 (8%). The length of stay in the PICU (PICU LOS) was examined in 12 (92%) studies; 3 (50%) studies showed a decrease in PICU LOS and 9 (50%) studies showed no change.

Implementation strategies were described in 11 (85%) studies and predominantly included educational modules, visual aids, and bedside local champions. The majority of implementation strategies center around educational efforts, despite the fact that educational efforts are known to be relatively weak interventions (17). Some studies included the usage of in-person champions for just-in-time decision support, though these were temporary interventions and did not report sustained impact (6–10, 12, 18). Furthermore, although most of the studies describe their implementation strategy, in very few reports, a compliance metric demonstrating the degree of implementation success. This makes interpreting the impact of the SA protocol on the outcome difficult, as “unsuccessful” outcomes may reflect low compliance rather than ineffective intervention. Despite the fact that the majority of the studies did not demonstrate improvements in patient-centered outcomes, none of the studies analyzed the reasons why the implementation was not successful.

Randomized Evaluation of Sedation Titration for Respiratory Failure (RESTORE), a multicenter unblinded cluster-randomized trial that included 31 PICUs in the United States (18), was the largest study in our review. The RESTORE study intervention consisted of standard pain, sedation and withdrawal score assessments, nurse-implemented goal-directed sedation protocol, and daily extubation readiness assessments. The primary outcome was the duration of mechanical ventilation, measured as ventilator-free days up to 28 days (VFD28). Secondary outcomes included PICU and hospital LOS, sedation-related adverse events, sedative exposure, and occurrence of iatrogenic withdrawal. Compliance with the protocol ranged from 71 to 100% depending on the study site. The primary outcome of VFD28 was not statistically significant between the intervention group and the control group.

The 2022 Society of Critical Care Medicine Clinical Practice Guidelines on Prevention and Management of Pain, Agitation, Neuromuscular Blockade, and Delirium in Critically Ill Pediatric Patients With Consideration of the ICU Environment and Early Mobility (PANDEM guidelines) reviewed many of these studies (2). However, given the heterogeneity of the data, the only strong recommendations related to sedation management were utilization of the comfort behavior scale (COMFORT-B) score or State Behavioral Scale to assess the level of sedation in mechanically ventilated patients and usage of dexmedetomidine as the primary sedative class specifically in critically ill pediatric post-operative cardiac surgical patients with expected early extubation. Utilization of protocolized sedation is listed as a suggestion with conditional strength and low quality of evidence.

## Outcomes Related to Sedation-Analgesia Practice

Providers in the PICU must find the balance between providing comfort to critically ill children who underwent invasive interventions while minimizing short- and long-term consequences of the sedative and analgesia medications. In the short term, many sedative agents may cause hypotension, bradycardia, and respiratory depression, which are managed in the PICU as anticipated adverse reactions but may prolong LOS. Furthermore, the consequences of lengthy sedation can include delirium, physical deconditioning, and ICU myopathy, which may not only prolong ICU and overall hospital LOS but also have longer-term impacts on mental health (3, 19, 20). The strong association of benzodiazepines with PICU delirium should prompt future studies of the impact of benzodiazepine-sparing regimens on PICU delirium and is one of the priorities of the PANDEM guideline (2–4). PICU delirium is a significant morbidity and a potentially modifiable factor that may impact the long-term outcomes related to a given SA protocol.

As we have reviewed, existing studies on SA protocols show promise in improving patient outcomes, though there are still gaps to address. Future directions for SA research in the PICU include optimizing study design, a focus on strategic implementation of interventions, efforts to sustain interventions over time, and inclusion of patient-centered outcomes, such as the prevalence of ICU delirium, long-term neurocognitive function, and behavioral health issues. A study examining long-term neurocognitive outcomes after ICU discharge is currently being designed by the RESTORE cognition study investigators (21). All of these priorities are essential to a meaningful and impactful practice change that becomes ingrained in PICU culture with long-term patient benefits supported by evidence-based medicine. SA protocols are just one piece of the ICU Liberation Bundle and would likely be strengthened if implemented with other practices, such as early mobility, routine extubation readiness assessment, and family engagement.

## Challenges With Implementation of Sedation and Analgesia Regimens

Challenges in implementing optimal SA in the PICU include patient heterogeneity (in pathology and weight-based dosing strategies), inability to engage non-verbal patients with non-pharmacologic interventions, concerns about medication effects on long-term neurocognitive outcomes, and the need to balance the depth of sedation with patient safety (such as unplanned extubations or line/tube dislodgment events) (12). Protocolized titration of SA requires reliable and reproducible bedside tools to assess sedation/comfort, analgesia, withdrawal, and delirium. Lack of acceptance for changes to SA regimens might stem from safety concerns with patients at a lighter level of sedation, distrust of newer sedative agents (e.g., dexmedetomidine vs. midazolam), or mobilization of ventilated patients.

The evidence for best SA practices remains scarce with respect to patient-centered outcomes, which may limit provider buy-in, even in the context of increased interest or motivation to change practice. The lack of newer effective drugs with acceptable pediatric safety profiles limits our choice of sedative agents. For example, the use of propofol as a long-term sedative agent is declined in children over the past decade due to concerns for propofol infusion syndrome and increased mortality (22). Midazolam has been associated with an increased risk of ICU delirium and, therefore, potential accrual of long-term morbidity (3, 4, 23). Furthermore, although a number of studies have demonstrated safety in using dexmedetomidine as a primary sedative agent, the adoption of dexmedetomidine as a primary sedative in pediatric critical care is still lagging (6, 24–26). Of the 11 studies reviewed above, only three (27%) utilized dexmedetomidine as a first-line sedative agent (6, 8, 13).

Additionally, most of the interventions in this cohort relied on weak implementation methods, such as educational modules. Several studies recognized the importance of providing bedside clinical decision support (CDS), particularly in the early phase post-implementation, to ensure compliance and sustainability beyond the immediate implementation period (7, 10, 12). However, compliance is rarely measured and only commented upon in three studies (6, 7, 18).

Further barriers in implementation include cultural context barriers, i.e., readiness of the local environment for change, as well as other practical limitations, such as resource requirements, staffing models, lack of PICU or institutional leadership investment, and lack of effective teamwork and collaboration skills (27, 28). These context barriers are rarely assessed or discussed in research studies, yet present significant impediments to successful implementation.

## Using Implementation Science for Studies Examining the Impact of Best Practices in Pediatric Critical Care

The studies of SA regimen efficacy in the PICU highlight an important gap in IS in pediatric critical care. IS addresses the effective translation of evidence-based guidelines into bedside practice and is an emerging field of study in critical care (28). Specifically, it includes both the implementation of systemic models and research to understand the performance of the implementation (28).

In pediatric critical care, barriers to effective implementation of new guidelines are multifactorial and span different levels of the healthcare delivery system. A recent study using the integrated Promoting Action on Research Implementation in Health Services (iPARIHS) framework across 58 professionals in 8 United States PICUs utilized structured interviews to examine barriers, facilitators, and processes for change (29). Common themes included complex multiprofessional teams, high-stakes work at near-capacity, and a need for clear evidence as a motivator to integrate change into an already busy workflow. These factors impact the entire change process that includes planning, deciding to adopt change, implementation, facilitator, and sustainability. However, such factors are largely qualitative and difficult to assess in a rigorous quantifiable manner.

## Future Directions – Implementation Science Methodology

In addition to continuing clinical research studies targeted at understanding best SA practices in pediatrics, there should be a parallel effort to specifically examine the adoption and sustenance of the intervention using IS-specific methodology. In addition to the development of an evidence-based intervention, strategically ensuring the successful implementation and sustainment of the intervention is critical to short-term and long-term success. Successful implementation may require effective education, ongoing just-in-time CDS, continuous feedback and evaluation, and strategic planning based on local contextual factors. IS seeks an understanding of why or how an intervention is successful. For example, although the comprehensive ICU Liberation Bundle highlights guidelines related to early mobility, SA practices, and daily extubation readiness assessment for improving patient outcomes, successful implementation has not been consistently demonstrated, and current investigations focus on barriers, such as culture change (19, 27, 28, 30).

Implementation science methodology includes tools, such as implementation mapping, traditional quality improvement (QI) tools, education, and concept mapping (28, 31). Implementation mapping is a process that identifies determinants of implementation (i.e., barriers and facilitators), which are then “mapped” onto specific strategies to address implementation barriers (28). This is similar to other QI strategies that can be utilized, such as key driver diagrams, stakeholder analysis, cause-and-effect diagrams, and process mapping (32). Furthermore, care delivery in the ICU is a team-based approach. This means that specific strategies in ICU implementation should include promoting team-based and patient-centered care. Patient-centered care should be structured based on guidelines but flexible enough to be tailored to each case depending on just-in-time data input.

A recent review provides an overview of the associated theories, models, and frameworks of IS (33). The authors identified six broad determinants of successful implementation, which are as follows: (1) the implementation object, (2) the user/adopter (e.g., healthcare providers), (3) the end user (e.g., patients), (4) the context, (5) the strategy, and (6) the outcome. Traditional research papers often lack a systematic assessment of the context and strategy. In this case, the context may refer less to the type of clinical environment in which the study is performed and more to the social/cultural factors that affect implementation, representing both potential barriers and unique resources. The context analysis is vital for successful implementation, as the knowledge of available resources and known barriers may allow for the crafting of a more targeted and effective strategy. For example, if a barrier to implementation is due to staffing limitations, the mitigation strategy would be different than if the barrier is due to inherent resistance to change. For the former, leveraging alternative resources (e.g., incentive structures for program participation) may be effective, whereas for the latter, a sequential roll-out with early adopters to demonstrate feasibility and success may be more effective in creating change. In the SA papers reviewed (Table 1), none of the studies incorporated a discussion on the assessment of local barriers and resource/barrier-specific strategies for implementation. It is generally assumed that the relevant barrier is a knowledge gap, and therefore, the majority of the center of intervention solely around education. As a comparison, a recent study on implementing blood transfusion recommendations in PICUs incorporated an assessment of potential barriers and then a description of specific barrier-targeting strategies prior to implementation of their intervention (34). Assessment of barriers and resources can be performed with qualitative interviews, structured focus groups, surveys, and stakeholder analyses, which are commonly used tools in quality and process improvement research (29, 32).

Another potentially useful tool for IS is the Dissemination and Implementation (D&I) Models in Health Research and Practice available through the National Institutes of Health (35). The D&I Models Webtool includes a broad framework for project planning: Plan, Select (D&I Models), Combine (D&I Models), Adapt, Use, and Measure. The webtool also includes instructions and examples for creating logic models for planning interventions. Broad categories addressed in the logic model include the dynamic context in which the project is occurring within, the problem being addressed, the evidence behind the intervention, strategies for D&I, short- and long-term outcomes, mediators of the D&I process (e.g., context), and sustainability infrastructure. Again, context and strategy are critical components of this logic model, highlighting the importance of this assessment in IS.

Since many protocols and materials rely on team-based approaches, educational material should emphasize the role clarity of team members, as well as identify and employ specific skills and knowledge unique to each team member. This requires an interdisciplinary approach at all stages of implementation, from intervention design to execution to auditing, maintenance, and accountability (36). Common barriers include changing ICU culture, specifically, potential changes in workload, such as needing increased staffing to facilitate early mobility with minimizing sedation, or changes in autonomy when transitioning from physician-driven to nurse-driven sedation plans (28). However, culture change is often difficult to institute and even more difficult to measure. Key components to influence culture change involve buy-in from all levels, such as leadership advocacy, frontline provider champions, and patient and family engagement (28). Furthermore, since IS typically involves the application of evidence-based practices to all patients, large randomized control trials may not be feasible as the study design of choice. However, as evidenced by the strength of recommendations from the PANDEM guidelines, rigorous research methodology is still required for the assessment of meaningful interventions that affect relevant outcomes. Researchers should consider other quasi-experimental research designs, such as the interrupted time series (ITS) design (37). The ITS study design affords the added benefit of visualizing any potential secular trends over time while simultaneously utilizing segmented regression analysis for rigorous statistical processing. The ITS design is an emerging study design of choice in IS that is more rigorous than simple pre-post implementation studies. There is also potential feasibility in using difference-in-differences analysis with the ITS design that uses a contemporary control group in the analysis of the intervention (37).

## Conclusion: A Proposed Framework for Studies Examining the Impact of Sedation and Analgesia Regimens

**FIGURE 1:**
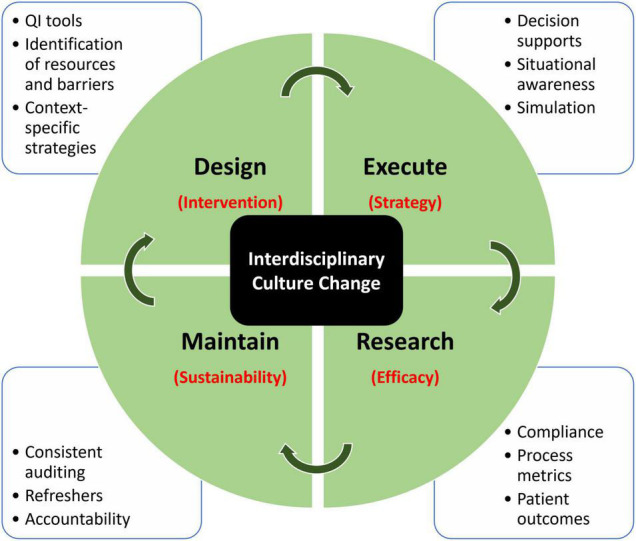
Proposed model of implementation research using a learning healthcare model. Each quadrant domain addresses a specific determinant (in red), which cohesively addresses the largest underlying challenge, which is interdisciplinary culture change. Surrounding each domain describes the tools that can be employed to address that specific component.

There is heightened interest in employing best practices related to SA regimens in pediatric critical care. There are several studies that have examined the role of evidence-based novel SA regimens in the PICU population, and this area of research has the promise to achieve improvements in patient outcomes. The existing literature on the subject could be significantly enhanced by emphasis on the systematic implementation of the interventions. Research in SA protocol implementation is met with numerous challenges (Figure 1). The intervention design requires consideration of medication choice and objective scoring systems. Understanding the efficacy of the intervention requires rigorous research methodology and thoughtful strategies to execute change. Lastly, even with successful implementation, maintaining sustainability has been an additional challenge (38). When healthcare systems build a new process for implementation, metrics examining compliance, process efficiency, and ongoing maintenance of the protocol should be prioritized. Central to this is institutional cultural alignment and a culture of shared responsibility. This describes components of a learning healthcare system that is better equipped for investigating, informing, instituting, and maintaining continual change (39). Taking all of the above into consideration, we recommend an interdisciplinary, data-driven, learning healthcare model to tackle IS and SA issues in pediatric critical care (Figure 1). The model incorporates components of Design, Educate, Research, and Maintain to highlight important components in the cycle of implementation. Further attention should be given to study the final step in the care delivery process using IS tools (40). The propagation of the implementation research framework and theory has not yet been systematically adopted in critical care research. However, critical care-specific IS training programs, as well as funding agencies, have recently been created (28). Future studies in SA practices in pediatrics should incorporate attention to methodology and data analytics specific to the IS step of the care delivery process, such as the context assessment of resources and barriers, and context-specific strategy planning. For example, identification of barriers and mapping of specific strategies to address each barrier should be included, and this should take place during the design phase of the implementation cycle. The full potential of basic science, clinical, and translational research can only be realized when we successfully jump the implementation hurdle and close the gap between evidence-based medicine and bedside practices in order to disseminate the best quality of care to all our patients (40).

## Author Contributions

YY and NM conceived the design of the manuscript. YY performed the review of the studies and prepared the manuscript draft. AG, KM, and NM reviewed and edited the manuscript. All authors approved the final version of the manuscript.

## Conflict of Interest

The authors declare that the research was conducted in the absence of any commercial or financial relationships that could be construed as a potential conflict of interest.

## Publisher’s Note

All claims expressed in this article are solely those of the authors and do not necessarily represent those of their affiliated organizations, or those of the publisher, the editors and the reviewers. Any product that may be evaluated in this article, or claim that may be made by its manufacturer, is not guaranteed or endorsed by the publisher.
